# Bendamustine Associated with Irreversible Ascending Paralysis

**DOI:** 10.1155/2013/931519

**Published:** 2013-02-27

**Authors:** Ashraf Alhafez, Omar S. Aljitawi, Tara L. Lin, Siddhartha Ganguly, Sunil Abhyankar, Joseph P. McGuirk

**Affiliations:** ^1^Internal Medicine Department, University of Kansas Medical Center, Kansas City, KS 66160, USA; ^2^Blood and Marrow Transplant Program, University of Kansas Medical Center, 2330 Shawnee Mission Pkwy, Westwood, Kansas City, KS 66205, USA

## Abstract

Bendamustine is an alkylating agent currently used in the treatment of lymphoproliferative disorders. Many adverse effects, including a rare case of reversible neurotoxicity, have been reported in association with bendamustine. Herein, we report the first case of irreversible ascending paralysis related to bendamustine.

## 1. Introduction

Bendamustine is a nitrogen mustard derivative containing a benzimidazole ring used in the treatment of chronic lymphocytic leukemia [1], relapsed indolent non-Hodgkin lymphoma (NHL) [2, 3], follicular lymphoma [4], multiple myeloma [5, 6], and mantle cell lymphoma [7]. Originally it was developed by Ozegowski and Krebs in East Germany [8]. In 2008, the Food and Drug Administration approved bendamustine for use in the treatment of chronic lymphocytic leukemia [9] and later for the treatment of indolent B-cell non-Hodgkin lymphoma [10]. Reported bendamustine related adverse effects have been limited to hematologic and gastrointestinal toxicities [11], fever, headache [12], and peripheral neuropathy [13]. In 2009, Cheson and Kroll reported an unusual case of reversible neurotoxicity felt to be secondary to bendamustine treatment [14]. The patient was a 63-year-old man who developed lower extremity numbness after treatment with bendamustine, which rapidly progressed over 1-2 weeks to include his entire legs, buttocks, and groins, and was associated with bowel and bladder incontinence, generalized lower extremity weakness, and severe altered mental status. Despite extensive investigation, no other etiology could be identified to explain his neurologic symptoms.

Herein, we report another case of neurotoxicity following bendamustine treatment; however, in our case the course was irreversible. 

## 2. Case Report

The patient is a 65-year-old female who was diagnosed in 2000 with non-Hodgkin lymphoma for which she had received chemotherapy and subsequently received an autologous transplant followed by an allogeneic transplant in 2006 for relapsed disease. She had a long history of chemotherapy induced peripheral neuropathy, for which she was on gabapentin. After transplant, she developed chronic graft versus host disease (GVHD). Despite that, she relapsed again in 2008 and was treated with bendamustine (90 mg/m^2^ days 2 and 3) and rituximab (375 mg/m^2^). Following two cycles of bendamustine and rituximab she achieved complete remission, but the third cycle was held due to delayed blood count recovery. 

Two months following her last cycle of bendamustine and rituximab, she developed lower extremity weakness that progressed to generalized weakness. This weakness was significant enough that she sustained several falls and then became bedbound. Her physical exam was remarkable for symmetrical upper extremity weakness with 4/5 strength, bilateral lower extremity weakness with 3/5 strength, 2-3/4 deep tendon reflexes, and no sensory deficit. Magnetic resonance imaging of the head showed generalized atrophy, small vessel ischemic disease, and an old right occipital lobe infarct. There was no acute intracranial process to explain her weakness. She also had magnetic resonance imaging of the cervical, thoracic, and lumbar spines, which were negative for any lesions that could explain her symptoms. Serum B12, copper, methylmalonic acid, and homocysteine levels were all within normal limits. Lumbar puncture was done and cerebrospinal fluid examination was unremarkable (glucose 70, protein 61, and lactate dehydrogenase 23) with no evidence of oligoclonal bands, malignant cells, or cryptococcus. CSF fluid studies were negative for JC virus, Epstein-Barr virus, Human herpesvirus 6, and cytomegalovirus by molecular tests. Her generalized weakness progressed further and then she developed visual deficits, floaters, and transient black spots in her left eye visual field. Ophthalmology service was consulted and it was felt that her visual symptoms were suspicious for nonarteritic ischemic optic neuropathy secondary to anemia and hypotension. 

The patient continued to experience severe deconditioning and weakness, and within 2 weeks she developed pain along her upper extremities exacerbated with touch and movements. This was associated with progressive decline in her mental status. Later, her overall pain including peripheral neuropathy pain worsened and she required increased narcotic use. At that juncture and in the setting of her progressive terminal disease, palliative care was consulted and comfort care measures were initiated until she expired two months from her initial presentation. Her immediate cause of death was ascending paralysis of central nervous system.

## 3. Discussion 

Bendamustine is regularly used to treat lymphoid malignancies. Reported side effects include hematological and gastrointestinal toxicities which are generally well tolerated [11]. Although, this drug has been used for forty years, neurotoxicity has never been reported as a side effect except for this case and the one described by Cheson and Kroll [14]. In both, the same regimen of bendamustine and rituximab was used prior to onset of symptoms and both patients presented in their seventh decade [15]. As in Cheson et al. case, we attribute the neurotoxicity in our case to bendamustine. Bendamustine shares a benzimidazole ring structure similar to the other purine analogs, and purine analogs have been implicated in cases of delayed onset neurotoxicity [16]. The neurotoxicity might also be indirectly related to bendamustine by causing immunosuppression, as prolonged decrease of CD4+ T-cell counts similar to that what occurs following treatment with fludarabine might contribute the bendamustine associated neurotoxicity [17]. One might argue that the neurotoxicity in our patient was related to rituximab use. However, the use of rituximab has been mainly incriminated in progressive multifocal leukoencephalopathy (PML) [18]. In our patient neuroimaging revealed no multifocal white matter process and cerebrospinal fluid examination was negative for JC virus, which makes the diagnosis of PML less likely [19]. In addition, rituximab has been used recently to treat immune-mediated neuropathies [20, 21]. Taking these arguments into consideration and since the extensive work up mentioned above did not identify any other etiology for this case of progressive weakness, we suspect this patient developing neurotoxicity secondary to bendamustine use. 

In conclusion, bendamustine should be considered in the differential diagnosis of progressive weakness and encephalopathy. Though this adverse event appears to be rare, it can lead to a devastating outcome like in our case. Further studies are needed to explore the mechanism underlying this toxicity.

## References

[1] Kath R, Blumenstengel K, Fricke HJ, Höffken K (2001). Bendamustine monotherapy in advanced and refractory chronic lymphocytic leukemia. *Journal of Cancer Research and Clinical Oncology*.

[2] Bremer K (2002). High rates of long-lasting remissions after 5-day bendamustine chemotherapy cycles in pre-treated low-grade non-Hodgkin’s-lymphomas. *Journal of Cancer Research and Clinical Oncology*.

[3] Heider A, Niederle N (2001). Efficacy and toxicity of bendamustine in patients with relapsed low-grade non-Hodgkin’s lymphomas. *Anti-Cancer Drugs*.

[4] Leonard JP, Martin P (2010). Novel agents for follicular lymphoma. *Hematology/American Society of Hematology Education Program*.

[5] Hata H (2011). Development of novel agents for multiple myeloma, now and the future. *Rinsho Ketsueki*.

[6] Cheson BD, Wendtner CM, Pieper A (2010). Optimal use of bendamustine in chronic lymphocytic leukemia, non-Hodgkin lymphomas, and multiple myeloma: treatment recommendations from an international consensus panel. *Clinical Lymphoma, Myeloma & Leukemia*.

[7] Chang JE, Kahl BS (2012). Bendamustine: more ammunition in the battle against mantle cell lymphoma. *Leukemia and Lymphoma*.

[8] Ozegowski WKD (1971). IMET, 3393, (-[1-methyl-5-bis-(b-chloroethyl)-amino-benzimidazolyl-(2)]-butyric acid hydrochloride, a new cytostatic agent from among the series of benzimidazole mustard compounds. *Zentralblatt für die Pharmazie, Pharmakotherapie und Laboratoriumsdiagnostik*.

[9] Cephalon Press Release (2008). *Cephalon Receives FDA Approval for TREANDA, a Novel Chemotherapy for Chronic Lymphocytic Leukemia*.

[10] Johansen KA, Schneider JF, McCaffree MA, Woods GL (2008). Efforts of the United States’ national marrow donor program and registry to improve utilization and representation of minority donors. *Transfusion Medicine*.

[11] Migliaccio AR, Adamson JW, Stevens CE, Dobrila NL, Carrier CM, Rubinstein P (2000). Cell dose and speed of engraftment in placental/umbilical cord blood transplantation: graft progenitor cell content is a better predictor than nucleated cell quantity. *Blood*.

[12] Friedberg JW, Cohen P, Chen L (2008). Bendamustine in patients with rituximab-refractory indolent and transformed non-Hodgkin's lymphoma: results from a phase II multicenter, single-agent study. *Journal of Clinical Oncology*.

[13] Höffken K, Merkle K, Schönfelder M (1998). Bendamustine as salvage treatment in patients with advanced progressive breast cancer: a phase II study. *Journal of Cancer Research and Clinical Oncology*.

[14] Cheson BD, Kroll ML (2009). Bendamustine induced neurotoxicity. *Clinical Advances in Hematology and Oncology*.

[15] Robinson KS, Williams ME, van der Jagt RH (2008). Phase II multicenter study of bendamustine plus rituximab in patients with relapsed indolent B-cell and mantle cell non-Hodgkin’s lymphoma. *Journal of Clinical Oncology*.

[16] Cheson BD, Vena DA, Foss FM, Sorensen JM (1994). Neurotoxicity of purine analogs: a review. *Journal of Clinical Oncology*.

[17] Kalaycio M, Wendtner CM (2009). What causes neurotoxicity after exposure to purine analogs?. *Clinical Advances in Hematology and Oncology*.

[18] Lysandropoulos AP, Du Pasquier RA (2010). Demyelination as a complication of new immunomodulatory treatments. *Current Opinion in Neurology*.

[19] Cinque P, Scarpellini P, Vago L, Linde A, Lazzarin A (1997). Diagnosis of central nervous system complications in HIV-infected patients: cerebrospinal fluid analysis by the polymerase chain reaction. *AIDS*.

[20] Motoyama R, Yamakawa K, Suzuki S, Kusunoki S, Tanaka M (2011). Rapid improvement by rituximab treatment in a case of demyelinating polyneuropathy with anti-myelin-associated glycoprotein antibody. *Rinsho Shinkeigaku*.

[21] Koike M, Sugimoto K, Tusui M, Yahata Y (2012). Successful treatment with rituximab in two cases of IgM-monoclonal gammopathy of undetermined significance (MGUS) neuropathy. *Rinsho Ketsueki*.

